# Correction: Synthesis of C14–C21 acid fragments of cytochalasin Z_8_*via anti*-selective aldol condensation and *B*-alkyl Suzuki–Miyaura cross-coupling

**DOI:** 10.1039/c8ra90018c

**Published:** 2018-02-21

**Authors:** Weiwei Han, Jinlong Wu

**Affiliations:** Laboratory of Asymmetric Catalysis and Synthesis, Department of Chemistry, Zhejiang University Hangzhou 310027 P. R. China wyz@zju.edu.cn +86-571-87953128 +86-571-87953128; College of Chemistry and Chemical Engineering, Xi’an Shiyou University Xi’an 710065 P. R. China vivien2014@xsyu.edu.cn +86-29-88382703

## Abstract

Correction for ‘Synthesis of C14–C21 acid fragments of cytochalasin Z_8_*via anti*-selective aldol condensation and *B*-alkyl Suzuki–Miyaura cross-coupling’ by Weiwei Han *et al.*, *RSC Adv.*, 2018, **8**, 3899–3902.

The authors regret that Jinlong Wu was not included in the author list in the original article. The correct list of authors and affiliations is presented above.

The Royal Society of Chemistry apologises for these errors and any consequent inconvenience to authors and readers.

## Supplementary Material

